# Supplementary material for: Implementation of prevention of mother-to-child transmission (PMTCT) in South Africa: outcomes from a population-based birth cohort study in Paarl, Western Cape
Source: BMJ Open. 2019 Dec 15;9(12):e033259. doi: 10.1136/bmjopen-2019-033259 (PMC6924830; doi:10.1136/bmjopen-2019-033259)
Supplement: Author's manuscript [file bmjopen-2019-033259.draft_revisions.pdf]

BMJ Open is committed to open peer review. As part of this commitment we make the peer review history of every article we publish publicly available.

When an article is published we post the peer reviewers' comments and the authors' responses online. We also post the versions of the paper that were used during peer review. These are the versions that the peer review comments apply to.

The versions of the paper that follow are the versions that were submitted during the peer review process. They are not the versions of record or the final published versions. They should not be cited or distributed as the published version of this manuscript.

BMJ Open is an open access journal and the full, final, typeset and author-corrected version of record of the manuscript is available on our site with no access controls, subscription charges or pay-per-view fees (http://bmjopen.bmj.com).

If you have any questions on BMJ Open's open peer review process please email info.bmjopen@bmj.com

Implementation of prevention of mother-to-child transmission (PMTCT) in South Africa: Outcomes from a population-based birth cohort study in Paarl, Western Cape

Journal:	*BMJ Open*
Manuscript ID	bmjopen-2019-033259
Article Type:	Research
Date Submitted by the Author:	28-Jul-2019
Complete List of Authors:	Pellowski, Jennifer; Brown University School of Public Health, Department of Behavioral and Social Sciences and International Health Institute<br>Wedderburn, Catherine; London School of Hygiene and Tropical Medicine, Department of Clinical Research<br>Stadler, Jacob; Red Cross War Memorial Children's Hospital, Department of Paediatrics and Child Health; South African Medical Research Council, Unit on Child and Adolescent Health<br>Barnett, Whitney; South African Medical Research Council, Unit on Child and Adolescent Health; Red Cross War Memorial Children's Hospital, Department of Paediatrics and Child Health<br>Stein, Dan; University of Cape Town, Department of Psychiatry & Mental Health; South African Medical Research Council, Unit on Risk and Resilience in Mental Disorders<br>Myer, Landon; University of Cape Town Faculty of Health Sciences, Division of Epidemiology and Biostatistics and Centre for Infectious Diseases Epidemiology and Research<br>Zar, Heather; Red Cross War Memorial Children's Hospital, Department of Paediatrics and Child Health; South African Medical Research Council, Unit on Child and Adolescent Health
Keywords:	Maternal medicine < OBSTETRICS, HIV & AIDS < INFECTIOUS DISEASES, South Africa, Implementation Science, cohort study, Global health

SCHOLARONE™
Manuscripts

Implementation of prevention of mother-to-child transmission (PMTCT) in South Africa: Outcomes from a population-based birth cohort study in Paarl, Western Cape

Authors:
Jennifer A. Pellowski[1,2] § *
Catherine J. Wedderburn[3,4] *
Jacob A. M. Stadler[4,5]
Whitney Barnett[4,5]
Dan J. Stein[6,7]
Landon Myer[8,9]
Heather J. Zar[4,5]

1. Department of Behavioral and Social Sciences, School of Public Health, Brown University, Rhode Island, USA
2. International Health Institute, School of Public Health, Brown University, Rhode Island, USA
3. Department of Clinical Research, London School of Hygiene & Tropical Medicine, UK
4. Department of Paediatrics and Child Health, Red Cross War Memorial Children's Hospital, University of Cape Town, Cape Town, South Africa
5. SA Medical Research Council, Unit on Child and Adolescent Health, University of Cape Town, South Africa
6. Department of Psychiatry & Mental Health, University of Cape Town, South Africa
7. South African Medical Research Council (SAMRC), Unit on Risk and Resilience in Mental Disorders, Cape Town, South Africa
8. Division of Epidemiology and Biostatistics, School of Public Health and Family Medicine, University of Cape Town, South Africa
9. Centre for Infectious Diseases Epidemiology and Research, School of Public Health and Family Medicine, University of Cape Town, South Africa

* J.A.P. and C.J.W. share first authorship.

§ Corresponding Author
Jennifer A. Pellowski, PhD, International Health Institute and Department of Behavioral and Social Sciences, Brown University School of Public Health, 121 South Main St. Providence, RI 02903. Email: Jennifer_pellowski@brown.edu; Phone: +1 (401) 863-6206

Short title/Running Head
Population-based PMTCT in South Africa

For peer review only

ABSTRACT

Objectives: Coverage of prevention of mother-to-child transmission (PMTCT) services in South Africa is variable. Identifying gaps in the implementation of these services is necessary to isolate steps needed to further reduce paediatric infections and eliminate transmission.

Setting: Two primary care clinics in Paarl, South Africa

Participants: 1225 pregnant women; inclusion criteria were 18 years or older, clinic attendance, and remaining in area for at least 1 year.

Methods: Data were collected through the Drakenstein Child Health Study, a population-based birth cohort in a peri-urban area of the Western Cape, South Africa. A combination of clinic records, hospital records, national database searches, and maternal self-report were collected during the study.

Results: Of the 1225 mothers enrolled in the cohort between 2012-2015, 260 (21%) were confirmed HIV-infected antenatally and 1 mother tested positive in the postnatal period. Of those with documentation (n=250/260, 96%), the majority (99%) received antiretroviral prophylaxis or therapy (ART) before labour, however, there was a high rate of defaulting noted during pregnancy (20%). All HIV-exposed infants with data received antiretroviral prophylaxis, 35% were exclusively breastfed till 6 weeks and 16% for 6 months. There were two cases of infant HIV-infection (0.8%), who were initiated on ART but had complicated histories.

Conclusions: Despite the low transmission rate in this cohort, reaching elimination will require further work and this study illustrates several areas to improve implementation of PMTCT services and reduce paediatric infections including retesting at-risk HIV negative mothers through the duration of breastfeeding, infant HIV testing at any admission in addition to routine testing, and improved counselling to prevent defaulting. Better data surveillance systems are essential for determining the implementation of PMTCT guidelines.

Key Words: Maternal health; HIV; South Africa; Implementation science; cohort studies; Global health

For peer review only

Strengths and limitations of this study

- This study quantifies the implementation and uptake of PMTCT guidelines in the current era identifying key gaps to inform how we may achieve very high coverage of a package of effective PMTCT interventions that will further reduce transmissions in high prevalence settings and achieve elimination.
- This research was conducted within a birth cohort study, whose primary aim was not to study HIV and PMTCT, providing a more natural study of implementation but with increased attention on data quality.
- The women included in these analyses were enrolled in a birth cohort study and may have been more uniquely motivated to attend clinics and engage in care than women not enrolled in the study, and thus these results may indicate a ‘best case’ scenario for engagement in PMTCT interventions.

For peer review only

INTRODUCTION

In the past decade there has been widespread progress globally in the prevention of mother-to-child transmission (PMTCT) of HIV and in 2014, the World Health Organization launched the call for elimination of mother-to-child transmission (MTCT) of HIV.(1) Countries must meet specific criteria to achieve elimination status, including ≤ 50 new paediatric infections per 100,000 live births. For countries with high prevalence of antenatal HIV, these targets are very challenging and will only be achieved with extremely low transmission rates requiring almost total coverage of a comprehensive package of PMTCT interventions.

South Africa has the highest number of HIV-infected people in the world with prevalence rates of up to 40% among public antenatal clinic attendees.(2) In 2010, the Western Cape government rolled out guidelines, which, based on a pregnant woman's clinical and immunological status, provided antiretroviral therapy (ART) for life or zidovudine (AZT) starting at 14 weeks gestation (Option A). In 2013, the Western Cape government rolled out Option B+, which provides ART for life for all pregnant women regardless of CD4 T-cell count. The first line regimen is triple therapy, comprising a non-nucleoside reverse-transcriptase inhibitor (NNRTI) backbone and two nucleoside reverse transcriptase inhibitors (NRTIs). Previous research, however, has shown that PMTCT service coverage in South Africa is variable, leading to missed opportunities for further reduction of transmission.(3)

A recent national evaluation(4) of the PMTCT programme showed a 6-week MTCT rate of 2.6% rising to 4.3% at 18 months postpartum, indicating the need to further explore the actual implementation of PMTCT programmes in South Africa. Lessons from the implementation and uptake of PMTCT guidelines in the current era are necessary to inform how we may achieve very high coverage of a package of effective PMTCT interventions that will further reduce transmissions in high prevalence settings and achieve elimination. In this report, we sought to quantify the implementation of PMTCT guidelines using data from the Drakenstein Child Health Study(5, 6), a population-based birth cohort in a peri-urban area of the Western Cape, South Africa.

METHODS

The Drakenstein Child Health Study recruited women during their second trimester of pregnancy (20-28 weeks gestation) from two community-based antenatal clinics in Paarl, South Africa.(5, 6) Women were eligible for the study if they attended one of the two study

For peer review only

clinics, planned to stay in the study area for at least one year, and were 18 years or older. All participants provided informed consent, prior to participation in any study activities, and were enrolled between 2012-2015.

Data Collection

Socio-demographic information was collected during the antenatal visit at 28-32 weeks gestation using structured interviews administered by trained study staff including: maternal age, education, marital status, employment and pregnancy planning.

HIV data were collected by triangulating clinic and hospital folder information and through self-report interviews completed with mothers during the antenatal period, at-birth and postpartum. Maternal HIV diagnosis was established at enrolment by self-report and confirmed during routine HIV testing of women in pregnancy as per the Western Cape PMTCT guidelines.(7, 8) All HIV-infected mothers were enrolled into the Provincial PMTCT programme. As the recruitment for the cohort spanned changes in the Provincial PMTCT guidelines, mothers were initiated on ART per guidelines at the time: Option A before 2013, moving to Option B+ in March 2013.

CD4 and viral load results were accessed from folder review and the online National Health Laboratory Service (NHLS) system. Where there was more than one result, the result closest to birth was used. ART was dichotomised into first and second/third line treatment, where the antiretroviral drug combinations relative to the guidelines at the time were used. Where available, data on regimen switching and defaulting during pregnancy were also obtained.

Infant PMTCT data was obtained from infant clinic folder review and HIV test results from the NHLS system. Infant feeding method was obtained through self-report from mothers at 6 weeks, 10-14 weeks, and 6 months postpartum as previously reported.(9) Ethics approval for this study was obtained through the Human Research Ethics Committee (HREC) of the Faculty of Health Sciences, University of Cape Town, Stellenbosch University, and the Western Cape Provincial Research committee.

Patient and Public Involvement

At the outset of the Drakenstein Child Health Study, close relationships were established with key stakeholders including Western Cape Government Health Department clinical and administrative staff, and community members. Patients were not involved in the design or recruitment of the study but at the 12 months postnatal study visit participants were

For peer review only

asked about their overall experiences with the study including experiences with study information, staff, procedures and how expectations about the study matched their experiences.(10) This information was used to reassess study protocols and to make alternations to improve study experience and participant satisfaction. Patients are not currently involved in dissemination plans, however, study findings are routinely fed back to the community and the Department of Health clinical and administrative staff.

RESULTS

A total of 1225 women enrolled in the study. Overall, 260 (21%) pregnant women were confirmed to be HIV positive through antenatal hospital and clinic chart reviews. At enrolment, HIV positive women were on average 29.1 years (SD=5.3), 25% were employed, 26% had completed secondary education, and 59% of pregnancies were unplanned.

Maternal Interventions

Of the known HIV positive women with available data, the majority (182/260, 70%) knew their HIV status prior to the study pregnancy (Table 1). One mother seroconverted in the postnatal period and thus, does not have antenatal PMTCT data. During the study pregnancy, most women had a CD4 T-cell count test (205/260; 79%) and the median CD4 count was 411 cells/mm^3 (interquartile range: 286-609) with 40% (82/205) having a CD4 of <350 cells/mm^3. Viral loads were available for 57% (148/260) of participants and most were undetectable (<40 copies/mL) (71%; 105/148). Prior to the 2013 guidelines, viral load testing was not routine during pregnancy and breastfeeding for those on lifelong ART.

The majority of women received antiretroviral drugs during pregnancy (248/250; 99%; 10 missing data), 39 (16%) were on PMTCT antiretroviral prophylaxis as per Option A (AZT), 195 (78%) were on first line triple ART and 14 (6%) on second/third line. Two participants (0.2%) were not on treatment in the antenatal period, but they received treatment during labour. There was evidence of defaulting from ART among 49 women (20%). Defaulters were not significantly different from non-defaulters by age, education, employment, or unplanned pregnancy.

Infant Interventions

Of the 260 mothers, 6 had miscarriages/in utero deaths, 13 were lost to follow up before birth, and there were 246 recorded live births with 4 sets of twins (Table 2). Of the live births, all infants with data were recorded as receiving prophylaxis; the majority of

For peer review only

infants (210/241; 87%) received nevirapine. Five infants (2%) did not have data postpartum and receipt of ART prophylaxis is unknown. Only 35% (82/235) of HIV positive mothers reported exclusive breastfeeding at 6 weeks postpartum and 16% (37/235) for 6 months. A concerning 5.5% of mothers reported mixed feeding at 6 weeks postpartum, rising to 12% by 3 months.

A total of 239 (97%) infants completed HIV PCR testing at 6-10 weeks postpartum, which were all negative. At 9 months, 196 infants had HIV testing (80%); of these, 195 (99.5%) were negative and 1 (0.5%) was equivocal with a repeat test that was negative. As part of the larger cohort study, 18-month tests were followed up prospectively. Of the children who were still in the cohort at 18 months (n=216), 192 had a negative 18-month test, 22 had a negative test post-cessation of breastfeeding or were formula-fed, 1 moved out of the area, and 1 tested equivocal and then confirmed HIV positive. There was also testing that occurred outside of these standard tests when infant symptoms warranted testing. During these tests, one infant tested HIV positive.

Mother-to-Child Transmission

Two infants in the cohort were confirmed HIV-infected, translating to a MTCT rate of 0.8% (2 infants/261 mothers). In one case the infant was born to a known HIV-infected mother with documented poor compliance to ART, poor maternal health and very poor social support structures. She booked at 18 weeks and had repeated viral load monitoring during pregnancy and postpartum, which showed an unsuppressed viral load throughout pregnancy and breastfeeding; she had already failed first line therapy. The child was born at 39 weeks gestation. The mother opted for breastfeeding and the infant was covered with nevirapine prophylaxis for the duration of exclusive breastfeeding, but compliance was questionable. Although the 6-10 weeks PCR and 9 months rapid test were negative, the infant had a positive rapid test at 18 months and was subsequently confirmed HIV positive by PCR and started on ART. However, both mother and child defaulted treatment and had to be restarted.

In the second case the mother tested HIV negative in the first trimester at booking (14 weeks) but subsequent testing was never done. The infant was born premature at 34 weeks gestation via vaginal delivery with a birth weight of 1.2kg and was not managed according to PMTCT protocols, as the child was considered unexposed. Exclusive breastfeeding was initiated, but the mother switched to formula feeding shortly after hospital discharge. Both mother and infant tested positive at 4 months of age when the infant was hospitalised with recurring pneumonia and was subsequently initiated on ART. This child was later diagnosed

For peer review only

with tuberculosis and then developed drug-induced hepatitis and required specialist treatment.

DISCUSSION

This study has shown a low rate of MTCT (0.8%) and reports on the entire PMTCT cascade with follow-up to 18 months. This may be due to the successful implementation of PMTCT guidelines in this peri-urban community, as demonstrated by the high rates of treatment in pregnant women and high rates of infant testing with low loss to follow up compared to what has been previously reported.(11) However, one-fifth of women defaulted from antiretroviral drugs at some point during pregnancy, indicating a need for improved adherence counselling.

Of the two HIV transmissions that occurred, both were identified after the 6-10 week infant PCR test. One transmission was to a known high-risk mother and the other the result of maternal seroconversion during the postnatal period while breastfeeding. These findings highlight several gaps to address to achieve PMTCT goals. These include the importance of recognising high-risk mothers (e.g. maternal poor adherence, first-line treatment failure, persistent unsuppressed viral loads) and providing increased support and careful follow-up of these mother-infant pairs throughout the duration of extended breastfeeding.(12) Secondly, these findings increase awareness of infants and mothers who are diagnosed following symptomatic presentation, and not via routine testing. These data reinforce the need to have a "belts and braces" approach to infant testing, including HIV testing at any infant admission in addition to routine testing.(13) Thirdly, it is important to retest HIV negative women during the breastfeeding period when women are at continued risk of transmitting acute infections to their infants. Fourthly, pre-exposure prophylaxis, an effective form of HIV prevention, should be considered for at-risk HIV negative breastfeeding mothers in combination with standard sexual risk reduction methods.

Data were obtained through a combination of clinic records, hospital records, national database searches, and maternal self-report. Despite this exhaustive search, there were some missing data, indicating issues with PMTCT surveillance systems, a key foundational factor to achieving elimination of transmission. Although South Africa's NHLS system tracks CD4 and viral load testing results, integrated data systems that track adherence to all PMTCT guidelines and can account for movement between clinical facilities are lacking. Furthermore, systems such as NHLS are themselves an underutilized tool for tracking individual adherence

For peer review only

to PMTCT guidelines. Training and resources are needed at the clinic level to identify and trace patients who may be missing these key PMTCT services.

There are some limitations to this study that should be considered. The women included in these analyses were enrolled in a birth cohort study and may have been more uniquely motivated to attend clinics and engage in care than women not enrolled in the study. Due to the eligibility criteria of the birth cohort study, participants had to present to the antenatal clinic in their second trimester. Thus, these results may indicate a 'best case' scenario for engagement in PMTCT interventions by excluding HIV positive mothers who are late presenters. Finally, this study, conducted in two communities in the peri-urban area of Paarl, Western Cape, may not be generalizable to other regions of the Western Cape or to other parts of South Africa.

CONCLUSIONS

Although South Africa does not currently meet the criteria for elimination of MTCT, this study demonstrates that attaining extremely high levels of PMTCT coverage to further reduce transmission rates in high prevalence regions may be within reach with re-testing, breastfeeding and ART adherence support, reinforced by improved data surveillance systems.

ACKNOWLEDGEMENTS

We greatly thank the families and children who participated in this study. We would like to thank the study staff in Paarl, the study data team and laboratory teams, the clinical and administrative staff of the Western Cape Government Health Department at Paarl Hospital and at the clinics for support of the study. In particular, we thank Nienke Schalij, Julia Bondar and Mary Familusi for all their contribution to this work. We acknowledge the advice from members of the study International Advisory Board and thank our collaborators.

CONTRIBUTORSHIP STATEMENT

All listed authors meet criteria for authorship. Individuals who contributed to this manuscript but do not meet the criteria for authorship are listed in the acknowledgements section. JP and CW conceptualized the analysis, analysed the data and wrote the first draft of the paper. HJZ is principal investigator of the parent study. DS and LM are coinvestigators as contributed to study design and implementation. CW, JS, and WB contributed to study implementation and data collection. All authors read and approved the final manuscript.

FUNDING

The study was funded by the Bill and Melinda Gates Foundation [OPP 1017641]. Additional support was provided by the Medical Research Council of South Africa (HJZ and DJS), the SAMRC National Health Scholars programme (WB), the Wellcome Trust through a

For peer review only

Research Training Fellowship [203525/Z/16/Z] (CJW) and the National Institute of Mental Health [K01 MH112443] (JAP).

COMPETING INTERESTS

None declared for any of the listed authors.

PATIENT CONSENT FOR PUBLICATION

Not required

ETHICS APPROVAL

Ethics approval for this study was obtained through the Human Research Ethics Committee (HREC) of the Faculty of Health Sciences, University of Cape Town, Stellenbosch University, and the Western Cape Provincial Research committee.

DATA SHARING STATEMENT

Deidentified participant data and syntax for these analyses are available from the Drakenstein Child Health Study data team (contact: Lesley.workman@uct.ac.za). Collaborations for the analysis of data are welcome. The DCHS has a large and active group of investigators and postgraduate students, and many have successfully partnered with researchers from other institutions. In particular, we encourage collaborations that lead to skills transfer and capacity building for postgraduate students. Researchers who are interested in datasets or collaborations can contact the PI, Heather Zar (heather.zar@uct.ac.za) with a concept note outlining the request. More information can be found on our website: http://www.paediatrics.uct.ac.za/scah/dclhs

For peer review only

REFERENCES

1. World Health Organization. Elimination of mother-to-child transmission (EMTCT) of HIV and syphilis: Global guidance on criteria and processes for validation2014 November 28, 2017.

2. Republic of South Africa Department of Health. The 2013 National Antenatal Sentinel HIV Prevalence Survey South Africa. 2015.

3. Woldesenbet S, Jackson D, Lombard C, et al. Missed Opportunities along the Prevention of Mother-to-Child Transmission Services Cascade in South Africa: Uptake, Determinants, and Attributable Risk (the SAPMTCTE). PLoS One. 2015;10(7):e0132425.

4. Goga A, Jackson D, Lombard C, et al. Highest risk of mother to child transmission of HIV or death in the first 6 months postpartum: results from 18 month follow-up of an HIV-exposed national cohort, South Africa. International AIDS Society Conference 2016; Durban, South Africa2016.

5. Stein DJ, Koen N, Donald KA, et al. Investigating the psychosocial determinants of child health in Africa: The Drakenstein Child Health Study. J Neurosci Methods. 2015;252:27-35.

6. Zar HJ, Barnett W, Myer L, Stein DJ, Nicol MP. Investigating the early-life determinants of illness in Africa: the Drakenstein Child Health Study. Thorax. 2015;70(6):592-4.

7. Health RoSADo. The South Africa Antiretroviral Treatment Guidelines 2015: PMTCT Guidelines: Revised March 2013. 2013.

8. Provincial Government of the Western Cape. The Western Cape Consolidated Guidelines for HIV Treatment: Prevention of Mother-to-Child Transmission of HIV (PMTCT), Children, Adolescents and Adults. 2015 (Amended Version). In: Department of Health HASTHD, editor. 2015.

9. Thomas E, Kuo C, Cohen S, et al. Mental health predictors of breastfeeding initiations and continuation among HIV infected and uninfected women in a South African birth cohort study. Prev Med. 2017;102(100-111).

10. Barnett WC, Brittain K, Sorsdahl K, Zar HJ, Stein DJ. Maternal participant experience in a South African birth cohort study enrolling health pregnant women and their infants. Philosophy, Ethics, and Humanities in Medicine. 2016;11(3).

11. Sibanda EL, Weller IVD, Hakim JG, Cowan FM. The magnitude of loss to follow-up of HIV-exposed infants along the prevention of mother-to-child HIV transmission continuum of care: a systematic review and meta-analysis. AIDS. 2013;27:2787-97.

12. Johnson LF, Stinson K, Newell M, et al. The contribution of maternal HIV seroconversion during late pregnancy and breastfeeding to mother-to-child transmission of HIV. J Acquir Immune Defic Syndr. 2012;59(4):417-25.

13. Kankasa C, Carter RJ, Briggs N, et al. Routine offering of HIV testing to hospitalized pediatric patients at University Teaching Hospital, Lusaka, Zambia: Acceptability and feasibility. . J Acquir Immune Defic Syndr. 2009;51(2):202-8.

For peer review only

Table 1. Maternal PMTCT results

Variable (n, %)		Total (n=260)
**HIV diagnosis time point †**		
	Before pregnancy	182 (71.4)
	During pregnancy	72 (28.2)
	After birth	1* (0.4)
**CD4 (during pregnancy)**		
	CD4 cells/mm^3 (median)(interquartile range)	411 (286 – 609)
	>=500 cells/ mm^3	78 (30.0)
	350-500 cells/mm^3	45 (17.3)
	<350 cells/mm^3	82 (31.5)
	No CD4 reported during pregnancy	55 (21.2)
**Viral load (VL) (during pregnancy)**		
	Virally unsuppressed (>=1000 copies/mL)	15 (5.8)
	VL detectable (>=40 - 1000 copies/mL)	28 (10.8)
	VL lower than detectable limit (<40 copies/mL)	105 (40.4)
	No VL reported during pregnancy	112 (43.1)
**Antiretroviral therapy (ART) initiation †**		
	Before pregnancy	98 (39.2)
	During pregnancy	150 (60.0)
	Received ARVs during labour only	2 (0.8)
**Antiretroviral regimen during pregnancy †**		
	PMTCT prophylaxis (AZT [zidovudine])	39 (15.6)
	1st line ART (triple therapy)	195 (78.0)
	2nd / 3rd line ART	14 (5.6)
	Not on treatment in the antenatal period	2 (0.8)
**ART regimen switched during pregnancy †**		10 (4.0)
**Evidence of defaulting any time during pregnancy †**		49 (19.6)

Footnotes:
Abbreviations: VL: Viral load; AZT: zidovudine; ART: Antiretroviral therapy

* This mother was diagnosed outside of the PMTCT pathway and as such she is not included in the other variables in this table.

† Percentages are calculated out of available data.
Missing data: HIV diagnosis time point n = 6; Antiretroviral therapy variables n = 10

For peer review only

Table 2. Child PMTCT results

Variable (n, %)	
**Infant birth outcomes**	**Total (n=265)**
Live births	246 (92.8)
Stillbirths / miscarriages / In utero deaths	6 (2.3)
Mothers lost to follow up before / at birth	13 (4.9)
**Infant PMTCT outcomes**	**Total (n=246)**
**Infant prophylaxis †**	
NVP prophylaxis	210 (87.1)
NVP + AZT prophylaxis	31 (12.9)
**Feeding method at 6 weeks †**	
Exclusively breastfeeding	82 (34.9)
Mixed feeding	13 (5.5)
Formula feeding	140 (59.6)
**Infant testing**	
**6-10 weeks postpartum**	
Positive	0 (0)
Negative	239 (97.2)
No test reported	7 (2.8)
**9 months postpartum**	
Positive	0 (0)
Negative	195 (79.3)
Equivocal, repeat test negative	1 (0.4)
No test reported	50 (20.3)
**HIV positive**	
Yes	2 (0.8) ‡

Footnotes:
Abbreviations: NVP: nevirapine; AZT: zidovudine

† Percentages are calculated out of available data for live births in follow up. Missing data: Infant prophylaxis n = 5; Feeding method n = 11

‡ 1 infant born to newly diagnosed HIV-positive mother

For peer review only

BMJ Open

Implementation of prevention of mother-to-child transmission (PMTCT) in South Africa: Outcomes from a population-based birth cohort study in Paarl, Western Cape

Journal:	*BMJ Open*
Manuscript ID	bmjopen-2019-033259.R1
Article Type:	Research
Date Submitted by the Author:	15-Oct-2019
Complete List of Authors:	Pellowski, Jennifer; Brown University School of Public Health, Department of Behavioral and Social Sciences and International Health Institute<br>Wedderburn, Catherine; London School of Hygiene and Tropical Medicine, Department of Clinical Research<br>Stadler, Jacob; Red Cross War Memorial Children's Hospital, Department of Paediatrics and Child Health; South African Medical Research Council, Unit on Child and Adolescent Health<br>Barnett, Whitney; South African Medical Research Council, Unit on Child and Adolescent Health; Red Cross War Memorial Children's Hospital, Department of Paediatrics and Child Health<br>Stein, Dan; University of Cape Town, Department of Psychiatry & Mental Health; South African Medical Research Council, Unit on Risk and Resilience in Mental Disorders<br>Myer, Landon; University of Cape Town Faculty of Health Sciences, Division of Epidemiology and Biostatistics and Centre for Infectious Diseases Epidemiology and Research<br>Zar, Heather; Red Cross War Memorial Children's Hospital, Department of Paediatrics and Child Health; South African Medical Research Council, Unit on Child and Adolescent Health
**Primary Subject Heading**:	HIV/AIDS
Secondary Subject Heading:	Global health, Paediatrics
Keywords:	Maternal medicine < OBSTETRICS, HIV & AIDS < INFECTIOUS DISEASES, South Africa, Implementation Science, cohort study, Global health

SCHOLARONE™
Manuscripts

Implementation of prevention of mother-to-child transmission (PMTCT) in South Africa: Outcomes from a population-based birth cohort study in Paarl, Western Cape

Authors:
Jennifer A. Pellowski[1,2] § *
Catherine J. Wedderburn[3,4] *
Jacob A. M. Stadler[4,5]
Whitney Barnett[4,5]
Dan J. Stein[6,7]
Landon Myer[8,9]
Heather J. Zar[4,5]

1. Department of Behavioral and Social Sciences, School of Public Health, Brown University, Rhode Island, USA
2. International Health Institute, School of Public Health, Brown University, Rhode Island, USA
3. Department of Clinical Research, London School of Hygiene & Tropical Medicine, UK
4. Department of Paediatrics and Child Health, Red Cross War Memorial Children's Hospital, University of Cape Town, Cape Town, South Africa
5. SA Medical Research Council, Unit on Child and Adolescent Health, University of Cape Town, South Africa
6. Department of Psychiatry & Mental Health, University of Cape Town, South Africa
7. South African Medical Research Council (SAMRC), Unit on Risk and Resilience in Mental Disorders, Cape Town, South Africa
8. Division of Epidemiology and Biostatistics, School of Public Health and Family Medicine, University of Cape Town, South Africa
9. Centre for Infectious Diseases Epidemiology and Research, School of Public Health and Family Medicine, University of Cape Town, South Africa

* J.A.P. and C.J.W. share first authorship.

§ Corresponding Author
Jennifer A. Pellowski, PhD, International Health Institute and Department of Behavioral and Social Sciences, Brown University School of Public Health, 121 South Main St. Providence, RI 02903. Email: Jennifer_pellowski@brown.edu; Phone: +1 (401) 863-6206

<u>Short title/Running Head</u>
Population-based PMTCT in South Africa

For peer review only

ABSTRACT

Objectives: Coverage of prevention of mother-to-child transmission (PMTCT) services in South Africa is variable. Identifying gaps in the implementation of these services is necessary to isolate steps needed to further reduce paediatric infections and eliminate transmission.

Setting: Two primary care clinics in Paarl, South Africa

Participants: 1225 pregnant women; inclusion criteria were 18 years or older, clinic attendance, and remaining in area for at least 1 year.

Methods: Data were collected through the Drakenstein Child Health Study, a population-based birth cohort in a peri-urban area of the Western Cape, South Africa. A combination of clinic records, hospital records, national database searches, and maternal self-report were collected during the study.

Results: Of the 1225 mothers enrolled in the cohort between 2012-2015, 260 (21%) were confirmed HIV-infected antenatally and 1 mother tested positive in the postnatal period. Of those with documentation (n=250/260, 96%), the majority (99%) received antiretroviral prophylaxis or therapy (ART) before labour, however, there was a high rate of defaulting from ART noted during pregnancy (20%). All HIV-exposed infants with data received antiretroviral prophylaxis, 35% were exclusively breastfed till 6 weeks and 16% for 6 months. There were two cases of infant HIV-infection (0.8%), who were initiated on ART but had complicated histories.

Conclusions: Despite the low transmission rate in this cohort, reaching elimination will require further work and this study illustrates several areas to improve implementation of PMTCT services and reduce paediatric infections including retesting at-risk HIV negative mothers through the duration of breastfeeding, infant HIV testing at any admission in addition to routine testing, and improved counselling to prevent defaulting from treatment. Better data surveillance systems are essential for determining the implementation of PMTCT guidelines.

Key Words: Maternal health; HIV; South Africa; Implementation science; cohort studies; Global health

For peer review only

Strengths and limitations of this study

- This study quantifies the implementation and uptake of PMTCT guidelines in the current era identifying key gaps to inform how we may achieve very high coverage of a package of effective PMTCT interventions that will further reduce transmissions in high prevalence settings and achieve elimination.
- This research was conducted within a birth cohort study, whose primary aim was not to study HIV and PMTCT, providing a more natural study of implementation but with increased attention on data quality.
- The women included in these analyses were enrolled in a birth cohort study and may have been more uniquely motivated to attend clinics and engage in care than women not enrolled in the study, and thus these results may indicate a 'best case' scenario for engagement in PMTCT interventions.

For peer review only

INTRODUCTION

In the past decade there has been widespread progress globally in the prevention of mother-to-child transmission (PMTCT) of HIV and in 2014, the World Health Organization launched the call for elimination of mother-to-child transmission (MTCT) of HIV.(1) Countries must meet specific criteria to achieve elimination status, including ≤ 50 new paediatric infections per 100,000 live births. For countries with high prevalence of antenatal HIV, these targets are very challenging and will only be achieved with extremely low transmission rates requiring almost total coverage of a comprehensive package of PMTCT interventions.

South Africa has the highest number of HIV-infected people in the world with prevalence rates of up to 40% among public antenatal clinic attendees.(2) In 2010, the Western Cape government rolled out guidelines, which, based on a pregnant woman's clinical and immunological status, provided antiretroviral therapy (ART) for life or zidovudine (AZT) starting at 14 weeks gestation (Option A). In 2013, the Western Cape government rolled out Option B+, which provides ART for life for all pregnant women regardless of CD4 T-cell count. The first line regimen is triple therapy, comprising a non-nucleoside reverse-transcriptase inhibitor (NNRTI) and two nucleoside reverse transcriptase inhibitors (NRTIs). Previous research, however, has shown that PMTCT service coverage in South Africa is variable, leading to missed opportunities for further reduction of transmission.(3)

A recent national evaluation(4) of the PMTCT programme showed a 6-week MTCT rate of 2.6% rising to 4.3% at 18 months postpartum, indicating the need to further explore the actual implementation of PMTCT programmes in South Africa. Lessons from the implementation and uptake of PMTCT guidelines in the current era are necessary to inform how we may achieve very high coverage of a package of effective PMTCT interventions that will further reduce transmissions in high prevalence settings and achieve elimination. In this report, we sought to quantify the implementation of PMTCT guidelines using data from the Drakenstein Child Health Study(5, 6), a population-based birth cohort in a peri-urban area of the Western Cape, South Africa.

METHODS

The Drakenstein Child Health Study recruited women during their second trimester of pregnancy (20-28 weeks gestation) from two community-based antenatal clinics in Paarl,

For peer review only

South Africa.(5, 6) Women were eligible for the study if they attended one of the two study clinics, planned to stay in the study area for at least one year, and were 18 years or older. All participants provided informed consent, prior to participation in any study activities, and were enrolled between 2012-2015.

Data Collection

Socio-demographic information was collected during the antenatal visit at 28-32 weeks gestation using structured interviews administered by trained study staff including: maternal age, education, marital status, employment and pregnancy planning.

HIV data were collected by triangulating clinic and hospital folder information and through self-report interviews completed with mothers during the antenatal period, at-birth and postpartum. Maternal HIV diagnosis was established at enrolment by self-report and confirmed during routine HIV testing of women in pregnancy as per the Western Cape PMTCT guidelines.(7, 8) All HIV-infected mothers were enrolled into the Provincial PMTCT programme. As the recruitment for the cohort spanned changes in the Provincial PMTCT guidelines, mothers were initiated on ART per guidelines at the time: Option A before 2013, moving to Option B+ in May 2013.

CD4 and viral load results were accessed from folder review and the online National Health Laboratory Service (NHLS) system. Where there was more than one result, the result closest to birth was used. ART was dichotomised into first and second/third line treatment, where the antiretroviral drug combinations relative to the guidelines at the time were used. Where available, data on regimen switching and treatment defaulting during pregnancy were also obtained. Defaulting was defined as an interruption (or discontinuation) of ART for at least a month during the index pregnancy.

Infant PMTCT data was obtained from infant clinic folder review and HIV test results from the NHLS system. Infant feeding method was obtained through self-report from mothers at 6 weeks, 10-14 weeks, and 6 months postpartum as previously reported.(9) Infant feeding was categorised into exclusive breastfeeding, formula feeding or mixed breastfeeding (breastfeeding plus formula milk and/or solids). N's, percentages, medians, and interquartile ranges are reported for all variables of interest. Ethics approval for this study was obtained through the Human Research Ethics Committee (HREC) of the Faculty of Health Sciences, University of Cape Town, Stellenbosch University, and the Western Cape Provincial Research committee.

For peer review only

Patient and Public Involvement

At the outset of the Drakenstein Child Health Study, close relationships were established with key stakeholders including Western Cape Government Health Department clinical and administrative staff, and community members. Patients were not involved in the design or recruitment of the study but at the 12 months postnatal study visit participants were asked about their overall experiences with the study including experiences with study information, staff, procedures and how expectations about the study matched their experiences.(10) This information was used to reassess study protocols and to make alternations to improve study experience and participant satisfaction. Patients are not currently involved in dissemination plans, however, study findings are routinely fed back to the community and the Department of Health clinical and administrative staff.

RESULTS

A total of 1225 women enrolled in the study. Overall, 260 (21%) pregnant women were confirmed to be HIV positive through antenatal hospital and clinic chart reviews (Figure 1). At enrolment, women living with HIV were on average 29.1 years (SD=5.3), 25% were employed, 26% had completed secondary education, and 59% of pregnancies were unplanned.

Maternal Interventions

Of the known women living with HIV with available data, the majority (182/260, 70%) knew their HIV status prior to the study pregnancy (Table 1). One mother seroconverted in the postnatal period and thus, does not have antenatal PMTCT data. During the study pregnancy, most women had a CD4 T-cell count test (205/260; 79%) and the median CD4 count was 411 cells/mm^3 (interquartile range: 286-609) with 40% (82/205) having a CD4 of <350 cells/mm^3. Viral loads were available for 57% (148/260) of participants and most were undetectable (<40 copies/mL) (71%; 105/148). Prior to the 2013 guidelines, viral load testing was not routine during pregnancy and breastfeeding for those on lifelong ART.

The majority of women received antiretroviral drugs during pregnancy (248/250; 99%; 10 missing data), 39 (16%) were on PMTCT antiretroviral prophylaxis as per Option A (AZT), 195 (78%) were on first line triple ART and 14 (6%) on second/third line. Two participants (0.2%) only received treatment during labour. There was evidence of defaulting

For peer review only - http://bmjopen.bmj.com/site/about/guidelines.xhtml

from ART among 49 women (20%). Defaulters were not significantly different from non-defaulters by age, education, employment, or unplanned pregnancy.

Infant Interventions

Of the 260 mothers, 5 had miscarriages/in utero deaths (including 1 set of twins), 11 were lost to follow up before birth, and there were 248 recorded live births with 4 sets of twins (Table 2). Of the live births, all infants with data were recorded as receiving prophylaxis; the majority of infants (211/242; 87%) received nevirapine. Six infants (2%) did not have data postpartum and receipt of ART prophylaxis is unknown. Only 35% (82/235) of mothers living with HIV reported exclusive breastfeeding at 6 weeks postpartum and 16% (37/235) for 6 months. A concerning 5.5% of mothers reported mixed feeding (breastfeeding plus formula milk and/or/solids) at 6 weeks postpartum, rising to 12% by 3 months.

A total of 239 (96%) infants completed HIV PCR testing at 6-10 weeks postpartum, which were all negative. At 9 months, 195 infants had HIV testing (78%); of these, 194 (99.5%) were negative and 1 (0.5%) was equivocal with a repeat test that was negative. As part of the larger cohort study, 18-month tests were followed up prospectively. Of the children who were still in the cohort at 18 months (n=220), 194 had a negative 18-month test, 22 had a negative test post-cessation of breastfeeding or were formula-fed, 3 moved out of the area, and 1 tested equivocal and then confirmed HIV positive. There was also testing that occurred outside of these standard tests when infant symptoms warranted testing. During these tests, one infant tested HIV positive.

Mother-to-Child Transmission

Two infants in the cohort were confirmed HIV-infected, translating to a MTCT rate of 0.8% (2 infants/261 mothers). In one case the infant was born to a known HIV-infected mother with documented poor compliance to ART, poor maternal health and very poor social support structures. She booked at 18 weeks and had repeated viral load monitoring during pregnancy and postpartum, which showed an unsuppressed viral load throughout pregnancy and breastfeeding; she had already failed first line therapy. The child was born at 39 weeks gestation. The mother opted for breastfeeding and the infant was covered with nevirapine prophylaxis however, it is unclear if this was for the duration of exclusive breastfeeding. Although the 6-10 weeks PCR and 9 months rapid test were negative, the infant had a positive rapid test at 18 months and was subsequently confirmed HIV positive by PCR and started on ART. However, both mother and child defaulted treatment and had to be restarted.

For peer review only

In the second case the mother tested HIV negative in the first trimester at booking (14 weeks) but subsequent testing was never done. The infant was born premature at 34 weeks gestation via vaginal delivery with a birth weight of 1.2kg and was not managed according to PMTCT protocols, as the child was considered unexposed. Exclusive breastfeeding was initiated, but the mother switched to formula feeding shortly after hospital discharge. Both mother and infant tested positive at 4 months of age when the infant was hospitalised with recurring pneumonia and was subsequently initiated on ART. This child was later diagnosed with tuberculosis and then developed drug-induced hepatitis and required specialist treatment.

DISCUSSION

This study has shown a low rate of MTCT (0.8%) and reports on the entire PMTCT cascade with follow-up to 18 months. This may be due to the successful implementation of PMTCT guidelines in this peri-urban community, as demonstrated by the high rates of treatment in pregnant women and high rates of infant testing with low loss to follow up compared to what has been previously reported.(11) However, one-fifth of women defaulted from antiretroviral drugs at some point during pregnancy, indicating a need for improved adherence counselling. Furthermore, nearly one-third of the sample had a CD4 T cell count of <350 with a large majority of women diagnosed pre-pregnancy, underscoring the importance of further focus on HIV care for women in general, outside of pregnancy.

Of the two HIV transmissions that occurred, both were identified after the 6-10 week infant PCR test. One transmission was to a known high-risk mother and the other to a mother diagnosed during the postnatal period. These findings highlight several gaps to address to achieve PMTCT goals. These include the importance of recognising high-risk mothers (e.g. maternal poor adherence, first-line treatment failure, persistent unsuppressed viral loads) and providing increased support and careful follow-up of these mother-infant pairs throughout the duration of extended breastfeeding.(12) Secondly, these findings increase awareness of infants and mothers who are diagnosed following symptomatic presentation, and not via routine testing. These data reinforce the need to have a “belts and braces” approach to infant testing, including HIV testing at any infant admission in addition to routine testing.(13) Thirdly, it is important to retest HIV negative women during later pregnancy, during labor, and during the breastfeeding period when women are at continued risk of transmitting acute infections to their infants. Fourthly, pre-exposure prophylaxis, an effective form of HIV prevention, should be considered for at-risk HIV negative breastfeeding mothers in

For peer review only

combination with standard sexual risk reduction methods. Finally, our sample had low rates of education and high rates of unplanned pregnancies, which are likely indicative of structural factors that may indirectly impede PMTCT efforts, underscoring the need to focus on broader contextual contexts in addition to clinical efforts.

Data were obtained through a combination of clinic records, hospital records, national database searches, and maternal self-report. Despite this exhaustive search, there were some missing data, indicating issues with PMTCT surveillance systems, a key foundational factor to achieving elimination of transmission. Although South Africa's NHLS system tracks CD4 and viral load testing results, integrated data systems that track adherence to all PMTCT guidelines and can account for movement between clinical facilities are lacking. Furthermore, systems such as NHLS are themselves an underutilized tool for tracking individual adherence to PMTCT guidelines. Training and resources are needed at the clinic level to identify and trace patients who may be missing these key PMTCT services.

There are some limitations to this study that should be considered. The women included in these analyses were enrolled in a birth cohort study and may have been more uniquely motivated to attend clinics and engage in care than women not enrolled in the study. Due to the eligibility criteria of the birth cohort study, participants had to present to the antenatal clinic in their second trimester. Thus, these results may indicate a 'best case' scenario for engagement in PMTCT interventions by excluding mothers living with HIV who are late presenters. Additionally, postnatal HIV testing of women who previously tested negative occurred outside of study procedures and was obtained through self-reports of significant maternal medical changes. Thus, the one mother who reported a postpartum diagnosis is potentially an underestimate for our cohort. Finally, this study, conducted in only two communities in the peri-urban area of Paarl, Western Cape, limiting our sample size of mothers living with HIV and the generalizability to other regions of the Western Cape or to other parts of South Africa. The Western Cape has some of the longest running PMTCT programs in SA and has some of the lowest MTCT rates in South Africa.(14) Our study highlights that even in areas where PMTCT programs are well functioning, there are still areas for improvement particularly in the context of elimination of MTCT.

CONCLUSIONS

Although South Africa does not currently meet the criteria for elimination of MTCT, this study demonstrates that attaining extremely high levels of PMTCT coverage to further reduce transmission rates in high prevalence regions may be within reach. This may be

For peer review only - http://bmjopen.bmj.com/site/about/guidelines.xhtml

achieved through re-testing, breastfeeding and ART adherence support, reinforced by improved data surveillance systems.

ACKNOWLEDGEMENTS

We greatly thank the families and children who participated in this study. We would like to thank the study staff in Paarl, the study data team and laboratory teams, the clinical and administrative staff of the Western Cape Government Health Department at Paarl Hospital and at the clinics for support of the study. In particular, we thank Nienke Schalij, Julia Bondar and Mary Familusi for all their contribution to this work. We acknowledge the advice from members of the study International Advisory Board and thank our collaborators.

CONTRIBUTORSHIP STATEMENT

All listed authors meet criteria for authorship. Individuals who contributed to this manuscript but do not meet the criteria for authorship are listed in the acknowledgements section. JP and CW conceptualized the analysis, analysed the data and wrote the first draft of the paper. HJZ is principal investigator of the parent study. DS and LM are coinvestigators as contributed to study design and implementation. CW, JS, and WB contributed to study implementation and data collection. All authors read and approved the final manuscript.

FUNDING

The study was funded by the Bill and Melinda Gates Foundation [OPP 1017641]. Additional support was provided by the Medical Research Council of South Africa (HJZ and DJS), the SAMRC National Health Scholars programme (WB), the Wellcome Trust through a Research Training Fellowship [203525/Z/16/Z] (CJW) and the National Institute of Mental Health [K01 MH112443] (JAP).

COMPETING INTERESTS

None declared for any of the listed authors.

PATIENT CONSENT FOR PUBLICATION

Not required

ETHICS APPROVAL

Ethics approval for this study was obtained through the Human Research Ethics Committee (HREC) of the Faculty of Health Sciences, University of Cape Town, Stellenbosch University, and the Western Cape Provincial Research committee.

DATA SHARING STATEMENT

Deidentified participant data and syntax for these analyses are available from the Drakenstein Child Health Study from the corresponding author. Collaborations for the analysis of data are welcome. The DCHS has a large and active group of investigators and postgraduate students, and many have successfully partnered with researchers from other institutions. In particular, we encourage collaborations that lead to skills transfer and capacity building for postgraduate students. Researchers who are interested in datasets or collaborations can

For peer review only

contact the PI, Heather Zar (heather.zar@uct.ac.za) with a concept note outlining the request. More information can be found on our website: www.paediatrics.uct.ac.za/scah/dclhs

Figure 1: Flow of participants through the Drakenstein Child Health Study

REFERENCES

1. World Health Organization. Elimination of mother-to-child transmission (EMTCT) of HIV and syphilis: Global guidance on criteria and processes for validation2014 November 28, 2017.
2. Republic of South Africa Department of Health. The 2013 National Antenatal Sentinel HIV Prevalence Survey South Africa. 2015.
3. Woldesenbet S, Jackson D, Lombard C, et al. Missed Opportunities along the Prevention of Mother-to-Child Transmission Services Cascade in South Africa: Uptake, Determinants, and Attributable Risk (the SAPMTCTE). PLoS One. 2015;10(7):e0132425.
4. Goga A, Jackson D, Lombard C, et al. Highest risk of mother to child transmission of HIV or death in the first 6 months postpartum: results from 18 month follow-up of an HIV-exposed national cohort, South Africa. International AIDS Society Conference 2016; Durban, South Africa2016.
5. Stein DJ, Koen N, Donald KA, et al. Investigating the psychosocial determinants of child health in Africa: The Drakenstein Child Health Study. J Neurosci Methods. 2015;252:27-35.
6. Zar HJ, Barnett W, Myer L, Stein DJ, Nicol MP. Investigating the early-life determinants of illness in Africa: the Drakenstein Child Health Study. Thorax. 2015;70(6):592-4.
7. Health RoSADo. The South Africa Antiretroviral Treatment Guidelines 2015: PMTCT Guidelines: Revised March 2013. 2013.
8. Provincial Government of the Western Cape. The Western Cape Consolidated Guidelines for HIV Treatment: Prevention of Mother-to-Child Transmission of HIV (PMTCT), Children, Adolescents and Adults. 2015 (Amended Version). In: Department of Health HASTHD, editor. 2015.
9. Thomas E, Kuo C, Cohen S, et al. Mental health predictors of breastfeeding initiations and continuation among HIV infected and uninfected women in a South African birth cohort study. Prev Med. 2017;102(100-111).
10. Barnett WC, Brittain K, Sorsdahl K, Zar HJ, Stein DJ. Maternal participant experience in a South African birth cohort study enrolling health pregnant women and their infants. Philosophy, Ethics, and Humanities in Medicine. 2016;11(3).
11. Sibanda EL, Weller IVD, Hakim JG, Cowan FM. The magnitude of loss to follow-up of HIV-exposed infants along the prevention of mother-to-child HIV transmission continuum of care: a systematic review and meta-analysis. AIDS. 2013;27:2787-97.
12. Johnson LF, Stinson K, Newell M, et al. The contribution of maternal HIV seroconversion during late pregnancy and breastfeeding to mother-to-child transmission of HIV. J Acquir Immune Defic Syndr. 2012;59(4):417-25.
13. Kankasa C, Carter RJ, Briggs N, et al. Routine offering of HIV testing to hospitalized pediatric patients at University Teaching Hospital, Lusaka, Zambia: Acceptability and feasibility. . J Acquir Immune Defic Syndr. 2009;51(2):202-8.
14. Barron P, Pillay Y, Doherty T, et al. Eliminating mother-to-child HIV transmission in South Africa. Bulletin of the World Health Organization. 2013;91:70-4.

For peer review only

Table 1. Maternal PMTCT results

Variable (n, %)		Total (n=260)
**HIV diagnosis time point †**		
	Before pregnancy	182 (71.4)
	During pregnancy	72 (28.2)
	After birth	1* (0.4)
**CD4 (during pregnancy)**		
	CD4 cells/mm^3 (median)(interquartile range)	411 (286 – 609)
	>=500 cells/ mm^3	78 (30.0)
	350-500 cells/mm^3	45 (17.3)
	<350 cells/mm^3	82 (31.5)
	No CD4 reported during pregnancy	55 (21.2)
**Viral load (VL) (during pregnancy)**		
	Virally unsuppressed (>=1000 copies/mL)	15 (5.8)
	VL detectable (>=40 - 1000 copies/mL)	28 (10.8)
	VL lower than detectable limit (<40 copies/mL)	105 (40.4)
	No VL reported during pregnancy	112 (43.1)
**Antiretroviral therapy (ART) initiation †**		
	Before pregnancy	98 (39.2)
	During pregnancy	150 (60.0)
	Received ARVs during labour only	2 (0.8)
**Antiretroviral regimen during pregnancy †**		
	PMTCT prophylaxis (AZT [zidovudine])	39 (15.6)
	1st line ART (triple therapy)	195 (78.0)
	2nd / 3rd line ART	14 (5.6)
	ARVs during labour only	2 (0.8)
**ART regimen switched during pregnancy †**		10 (4.0)
**Evidence of defaulting any time during pregnancy †**		49 (19.6)

Footnotes:
Abbreviations: VL: Viral load; AZT: zidovudine; ART: Antiretroviral therapy

* This mother was diagnosed outside of the PMTCT pathway and as such she is not included in the other variables in this table.

† Percentages are calculated out of available data.
Missing data: HIV diagnosis time point n = 6; Antiretroviral therapy variables n = 10

For peer review only - http://bmjopen.bmj.com/site/about/guidelines.xhtml

Table 2. Child PMTCT results

Variable (n, %)	
**Infant birth outcomes**	**Total (n=265)**
Live births	248 (93.6)
Stillbirths / miscarriages / In utero deaths	6 (2.3)
Mothers lost to follow up before / at birth	11 (4.2)
**Infant PMTCT outcomes**	**Total (n=248)**
**Infant prophylaxis †**	
NVP prophylaxis	211 (87.2)
NVP + AZT prophylaxis	31 (12.8)
**Feeding method at 6 weeks †**	
Exclusively breastfeeding	82 (34.9)
Mixed feeding	13 (5.5)
Formula feeding	140 (59.6)
**Infant testing**	
**6-10 weeks postpartum**	
Positive	0 (0)
Negative	239 (96.4)
No test reported	9 (3.6)
**9 months postpartum**	
Positive	0 (0)
Negative	194 (78.2)
Equivocal, repeat test negative	1 (0.4)
No test reported	53 (21.4)
**HIV positive**	
Yes	2 (0.8) ‡

Footnotes:
Abbreviations: NVP: nevirapine; AZT: zidovudine

† Percentages are calculated out of available data for live births in follow up. Missing data: Infant prophylaxis n = 6; Feeding method n = 13

‡ 1 infant born to newly diagnosed HIV-positive mother

For peer review only

For peer review only

Figure 1: Flow of participants through the Drakenstein Child Health Study

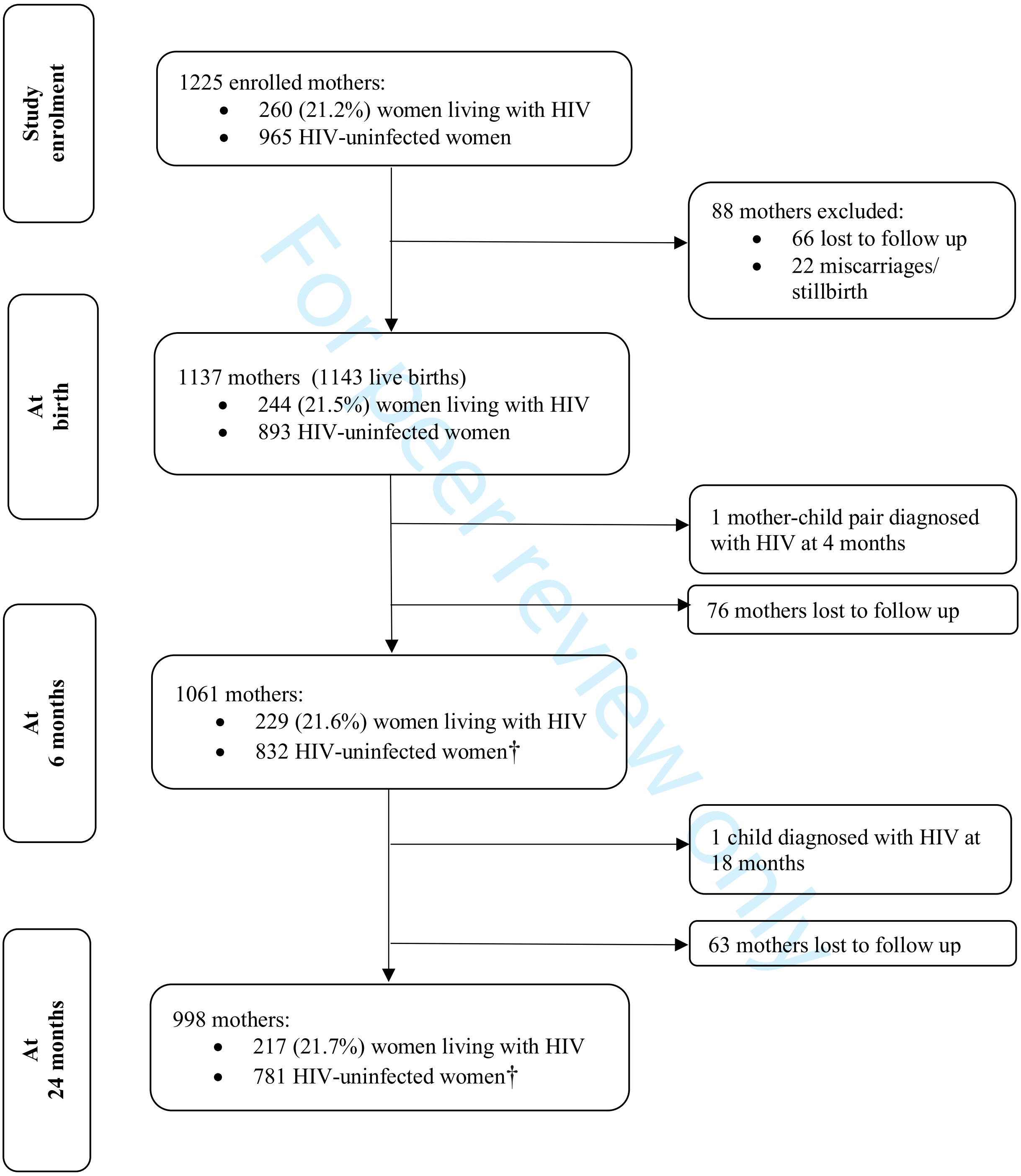

For peer review only

† Postnatal testing of women was not conducted through the cohort. Change in HIV status postpartum was reported by women during detailed data collection about changes in their medical history at multiple time points.

	Item No	Recommendation	Page Number
**Title and abstract**	1	(*a*) Indicate the study's design with a commonly used term in the title or the abstract	Page 1-2
		(*b*) Provide in the abstract an informative and balanced summary of what was done and what was found	Page 2
**Introduction**			
Background/rationale	2	Explain the scientific background and rationale for the investigation being reported	page 4
Objectives	3	State specific objectives, including any prespecified hypotheses	page 4
**Methods**			
Study design	4	Present key elements of study design early in the paper	pages 4-5
Setting	5	Describe the setting, locations, and relevant dates, including periods of recruitment, exposure, follow-up, and data collection	pages 4-5
Participants	6	(*a*) Give the eligibility criteria, and the sources and methods of selection of participants. Describe methods of follow-up	pages 4-5
		(*b*) For matched studies, give matching criteria and number of exposed and unexposed	N/A
Variables	7	Clearly define all outcomes, exposures, predictors, potential confounders, and effect modifiers. Give diagnostic criteria, if applicable	page 5
Data sources/ measurement	8*	For each variable of interest, give sources of data and details of methods of assessment (measurement). Describe comparability of assessment methods if there is more than one group	page 5
Bias	9	Describe any efforts to address potential sources of bias	N/A
Study size	10	Explain how the study size was arrived at	sub-study; main study (ref. Zar et al. 2015) contains these details

For peer review only

Quantitative variables	11	Explain how quantitative variables were handled in the analyses. If applicable, describe which groupings were chosen and why	page 5
Statistical methods	12	(*a*) Describe all statistical methods, including those used to control for confounding	page 5
		(*b*) Describe any methods used to examine subgroups and interactions	N/A
		(*c*) Explain how missing data were addressed	N/A descriptive paper
		(*d*) If applicable, explain how loss to follow-up was addressed	N/A descriptive paper
		(*e*) Describe any sensitivity analyses	N/A
**Results**			
Participants	13*	(a) Report numbers of individuals at each stage of study—eg numbers potentially eligible, examined for eligibility, confirmed eligible, included in the study, completing follow-up, and analysed	page 6-7
		(b) Give reasons for non-participation at each stage	N/A
		(c) Consider use of a flow diagram	N/A
Descriptive data	14*	(a) Give characteristics of study participants (eg demographic, clinical, social) and information on exposures and potential confounders	page 6
		(b) Indicate number of participants with missing data for each variable of interest	pages 6, 7
		(c) Summarise follow-up time (eg, average and total amount)	page 7
Outcome data	15*	Report numbers of outcome events or summary measures over time	Table 1 and 2
Main results	16	(*a*) Give unadjusted estimates and, if applicable, confounder-adjusted estimates and their precision (eg, 95% confidence interval). Make clear which confounders were adjusted for and why they were included	N/A descriptive paper

For peer review only

		(*b*) Report category boundaries when continuous variables were categorized	N/A descriptive paper
		(*c*) If relevant, consider translating estimates of relative risk into absolute risk for a meaningful time period	N/A descriptive paper
Other analyses	17	Report other analyses done—eg analyses of subgroups and interactions, and sensitivity analyses	N/A descriptive paper
**Discussion**			
Key results	18	Summarise key results with reference to study objectives	page 8
Limitations	19	Discuss limitations of the study, taking into account sources of potential bias or imprecision. Discuss both direction and magnitude of any potential bias	page 9
Interpretation	20	Give a cautious overall interpretation of results considering objectives, limitations, multiplicity of analyses, results from similar studies, and other relevant evidence	page 8-9
Generalisability	21	Discuss the generalisability (external validity) of the study results	page 9
**Other information**			
Funding	22	Give the source of funding and the role of the funders for the present study and, if applicable, for the original study on which the present article is based	Page 10

For peer review only
